# The Interplay between Synaptic Activity and Neuroligin Function in the CNS

**DOI:** 10.1155/2015/498957

**Published:** 2015-03-09

**Authors:** Xiaoge Hu, Jian-hong Luo, Junyu Xu

**Affiliations:** Department of Neurobiology, Key Laboratory of Medical Neurobiology of Ministry of Health, Zhejiang Province Key Laboratory of Neurobiology, Zhejiang University School of Medicine, Hangzhou, Zhejiang 310058, China

## Abstract

Neuroligins (NLs) are postsynaptic transmembrane cell-adhesion proteins that play a key role in the regulation of excitatory and inhibitory synapses. Previous *in vitro* and *in vivo* studies have suggested that NLs contribute to synapse formation and synaptic transmission. Consistent with their localization, NL1 and NL3 selectively affect excitatory synapses, whereas NL2 specifically affects inhibitory synapses. Deletions or mutations in NL genes have been found in patients with autism spectrum disorders or mental retardations, and mice harboring the reported NL deletions or mutations exhibit autism-related behaviors and synapse dysfunction. Conversely, synaptic activity can regulate the phosphorylation, expression, and cleavage of NLs, which, in turn, can influence synaptic activity. Thus, in clinical research, identifying the relationship between NLs and synapse function is critical. In this review, we primarily discuss how NLs and synaptic activity influence each other.

## 1. Introduction

Neuroligins (NLs) are postsynaptic transmembrane proteins [1] that feature a large extracellular acetylcholinesterase- (AChE-) like domain that lacks esterase activity, a single transmembrane domain, and a short cytoplasmic tail (c-tail) containing a type I PDZ-domain-binding motif [1, 2] that functions in intracellular protein-protein interactions and signaling processes. NLs have been identified in humans, rodents, chicken,* Drosophila melanogaster*, and* Caenorhabditis elegans* [1–6], and they have been linked to autism [7–15]. Whereas 5 NL isoforms are present in humans (NL1, NL2, NL3, NL4X, and NL4Y), only 4 are present in rodents [3, 4, 7, 16]. Despite high sequence conservation among distinct NL isoforms [2, 4], the subcellular distributions of NLs differ markedly: NL1 is predominantly localized at excitatory synapses, whereas NL2 exclusively localizes at inhibitory synapses [17–19]. Moreover, NL3 was reported to be localized at both excitatory and inhibitory synapses [20], and NL4 was localized to glycinergic synapses in a retina system [21].

NLs have been shown to bind, through their AChE-like domain, to the Laminin G/Neurexin/Sex Hormone Binding Globulin (LNS) domain of neurexins. When expressed in nonneuronal cells, NLs trigger presynaptic development by acting through neurexins [22]. Conversely, neurexins expressed in nonneuronal cells can cause NL aggregation and induce postsynaptic specializations [23].

Both NLs and neurexins feature a long extracellular domain and a short intracellular domain containing a PDZ-domain-binding motif, which is critical for synaptic protein recruitment (see [24, 25] for review). NLs have been shown to bind to the postsynaptic scaffold protein PSD-95 [26], which associates with ion channels [27] and neurotransmitter receptors, including NMDA receptors [28]. Furthermore, the expression level of PSD-95 affects the distribution of NLs at excitatory versus inhibitory synapses, as well as the balance between excitatory and inhibitory synapses [29, 30]. NL2 can also recruit GABA_A_ and glycine receptors by interacting with gephyrin and collybistin in a non-PDZ dependent manner [31].

NLs play a key role as mediators of synapse formation, as indicated by numerous* in vitro* studies in which their expression levels were manipulated [17, 23, 29, 30, 32, 33], and NL knockout (KO) or autism mutation knock-in (KI) animal models show deficits in synaptic transmission [21, 34–47]. In NL1/2/3 triple-KO mice, the total synapse number and ultrastructural synaptic features are normal, but these mice exhibit severe deficits in brainstem synaptic transmission [48]; this indicates that NLs are essential for proper synaptic function but not synapse morphology* in vivo*. NLs have also been widely reported to regulate NMDA and AMPA receptor (NMDAR and AMPAR) function [33, 39, 49–51] and to be involved in synaptic plasticity [35, 37, 50, 52–55].

NLs not only function in synapse formation and synaptic transmission, but are also influenced by synaptic activity [33, 56–62], especially in the case of NL1: synaptic activity can regulate NL1 surface expression [59–62]. Moreover, phosphorylation of endogenous NL2 was recently reported to disrupt NL2/gephyrin interaction and thereby downregulate GABAergic transmission [63].

The normal functioning of the brain relies on the proper assembly of neuronal circuits and the occurrence of synaptic transmission. Thus, it is crucial to understand how NLs regulate synapse function and how neuronal activity affects the regular functions of NLs. In this review, we primarily discuss—based on previous studies—the relationship between NLs and neuronal activity.

## 2. Function of Neuroligins in Synaptic Activity

### 2.1. NL1

Numerous* in vitro* and* in vivo* studies have implicated a critical role of NLs in synapse formation and synaptic transmission. In* in vitro* coculture systems, NLs expressed in HEK293 cells were shown to induce the formation of presynaptic structures in contacting axons [22]. The results of electrophysiological recordings of these artificial synapses support a key role of NLs in the formation of functional synapses [64, 65].

In the coculture system, NL1 overexpression on the surface of heterologous cells induced the clustering of both GAD-65 and vGluT [23]. Consistent with the synaptogenic activity of NLs observed in the coculture system, NL1 overexpression in cultured neurons enhanced both excitatory and inhibitory presynaptic differentiation [29]. Moreover, NL1 expression levels caused changes in the expression of both presynaptic and postsynaptic proteins [17, 29, 32, 33, 35, 37, 52–55, 57, 66–68] (Table 1, NL1). These changes were not limited to excitatory synapses: the formation of inhibitory synapses was also affected. Collectively, these results indicate that NL1 plays a role in both presynaptic and postsynaptic differentiation during synaptogenesis.

Altering NL1 expression levels not only induces changes in synapse density, but also affects synaptic transmission. NL1 overexpression was shown to markedly enhance basal synaptic transmission in cultured hippocampal neurons [29] (Table 2, NL1). Moreover, upon overexpression, NL1 specifically enhanced AMPAR-/NMDAR-mediated excitatory postsynaptic currents (EPSCs), but not inhibitory postsynaptic currents (IPSCs), in an NMDAR-dependent manner; this revealed a selective effect of NL1 on excitatory synaptic transmission [33] (Table 2, NL1). This could have been a sequential effect of altering receptor levels because the overexpression of NL1, but not NL2 or NL3, led to a substantial increase in NMDAR expression [53] (Table 1, NL1). Moreover, overexpression of a mutant form of NL1 (NL1 R473C), which was identified in patients with autism spectrum disorders (ASDs), led to a reduction in the number of excitatory synapses and also suppressed glutamatergic transmission [33] (Table 2, NL1). These results implied that changes in synaptic transmission could be an underlying cause of ASD.

The role of NL1 in excitatory synaptic transmission was also revealed using whole-cell patch clamp recordings in the CA1 area of acute hippocampal slices obtained from NL1 KO mice (Table 1, NL1). In these NL1 KO mice, the amplitude of NMDAR-mediated EPSCs, but not AMPAR-mediated EPSCs, was decreased, which resulted in a large reduction in the NMDA/AMPA ratio [33] and eliminated NMDAR-dependent long-term potentiation (LTP) [53]. Reintroduction of NL1 restored the NMDA/AMPA ratios and NMDAR-mediated EPSCs in NL1 KO slices [53]. These reported transmission defects could have resulted from the reduction of synaptosomal expression levels of AMPARs and NMDARs in NL1 KO mice [53]. More importantly, the detected electrophysiological defects could all be selectively alleviated by introducing a chimeric NL2 transplanted with NL1's extracellular AChE domain, but not by using a chimeric NL1 transplanted with NL2's AChE domain [53]; this indicated that the AChE domain of NL1 is necessary for normal synaptic transmission at the glutamatergic synapse, probably because of its involvement in the extracellular coupling of NL1 and NMDARs.

Although NL3 was also shown to be localized at excitatory synapses, the results of microRNA-mediated knockdown showed that NL1 specifically affects basal neuronal activity and LTP in the hippocampus [54] (Table 2, NL1 and NL3). The expression of chimeric forms of NL1 and NL3 in NL1–NL3 knockdown neurons showed that the difference between NL1 and NL3 detected in young hippocampal CA1 LTP were due to the extracellular B-site insertion of NL1 [54].

In addition to functioning in the hippocampus, NL1 plays a role in excitatory synaptic transmission in the amygdala. In the Sprague Dawley rats, acute silencing of endogenous NL1 in the amygdala by using lentiviral injection selectively lowered NMDAR-mediated EPSCs, and it also impaired LTP and weakened the storage of associative fear memory [35]. The NL1 KO mice also displayed a drastic reduction in NMDAR-mediated EPSCs at afferent inputs to the amygdala [50]. Moreover, in these mice, spike-timing-dependent LTP (STD-LTP) was markedly impaired at thalamic but not cortical inputs to the amygdala, where STD-LTP is NMDAR-independent [50]. Furthermore, reduced NMDA/AMPA ratios at corticostriatal synapses were shown to be the causes of repetitive behavior in NL1 KO mice, and the increased repetitive grooming behavior observed could be rescued in adult mice by administering the NMDAR-selective coagonist D-cycloserine [52]. Therefore, NL1 regulates excitatory synaptic transmission in an NMDAR-dependent and circuitry-specific manner.

In an NL1 transgenic mouse model, NL1 overexpression led to a sequential increase in spine and synapse number, excitatory/inhibitory (E/I) ratio, and synaptic transmission in the hippocampus [37] (Table 1, NL1). Intriguingly, both overexpression and downregulation of NL1 protein have been reported to impair LTP and also memory [35, 37]. It is possible that increased basal excitability and reduced ion channel conductivity which are observed in the NL1 transgenic mice and KO mice, respectively, both led to abnormal LTP expression and thereby affect learning and memory. Lastly, in* C. elegans*, NL1 and neurexin-1 control the kinetics of synaptic vesicle release through retrograde signaling at neuromuscular junctions [69].

### 2.2. NL2

Unlike NL1, NL2 is exclusively localized at inhibitory synapses [19]. Collectively,* in vitro* and* in vivo* studies have suggested a role of NL2 in synapse formation and function. Initially, in an* in vitro* coculture system, NL2 expressed in HEK293 cells was shown to induce the assembly of presynaptic structures in contacting axons [22]. Subsequently, patch clamp recordings detected GABAergic events in HEK293 cells coexpressing GABA_A_ receptors and NL2 in the coculture system, thus suggesting the functional reconstitution of GABAergic synapses [65].

In the coculture system, NL2 expressed in COS cells induced the clustering of both glutamatergic and GABAergic synaptic vesicles in contacting axons [23]. NL2, unlike NL1, associates with both PSD-95 and gephyrin, which are postsynaptic scaffolding proteins of excitatory and inhibitory synapses, respectively. When YFP-NL2 expressed in neurons was directly aggregated using beads coated with YFP antibodies, PSD-95 and gephyrin were coaggregated, with gephyrin being aggregated to a greater extent than PSD-95 [23]. The balance between excitatory and inhibitory synapses was also altered following PSD-95 overexpression or knockdown of gephyrin or PSD-95, which redistributed NL2 between inhibitory and excitatory synapses [30, 70].

In cultured hippocampal neurons, NL2 overexpression led to an increase in the number of both vGluT1 and vGAT puncta. The ratio of vGluT1/vGAT puncta was decreased considerably, indicating that NL2 influences the formation of both types of synapses, but preferentially affects inhibitory synapses [17] (Table 1, NL2). Moreover, when NL2 was knocked down using an NL2 shRNA, synaptic puncta and spine number were markedly lowered [17] (Table 1, NL2). In contrast to* in vitro* studies showing that NLs play a role in synapse formation, in NL2 KO mice, the number of asymmetric and symmetric synapses was unchanged [71]. This discrepancy could have arisen as a result of the difference between acute manipulation and chronic compensation.

Overexpressed NL2 caused a specific increase in IPSC amplitude [33]. Moreover, proline-directed phosphorylation of endogenous NL2 at S714 resulted in the recruitment of Pin1, a peptidyl-prolyl cis-trans isomerase, which negatively regulated NL2/gephyrin interaction and thereby downregulated GABAergic transmission [63]. Taken together, these findings suggest that NL2 regulates inhibitory synaptic transmission in a neuronal activity- and phosphorylation-dependent manner.

NL2 KO mice exhibit irregular breathing patterns much like NL1/2/3 triple-KO mice, which die within 24 h after birth because of breathing failure [48]. In various brain regions of NL2 KO mice, synaptic transmission and synapse formation were altered to distinct extents (Table 2, NL2). In acute slices obtained from the ventrolateral medulla of NL2 KO mice, inhibitory synaptic transmission was diminished at both GABAergic and glycinergic synapses, but no clear changes in synapse number were detected [31]. However, the hippocampal region of NL2 KO mice exhibited a marked and selective reduction in the density of inhibitory synaptic proteins vGAT, gephyrin, and GABAR *γ*2 subunit [31, 40], indicating specific effects of NL2 on inhibitory synapses. Moreover,* in vivo* recordings in NL2 KO mice showed drastically increased granule cell (GC) excitability in the dentate gyrus [40] (Table 2, NL2); here, GABAergic synaptic inhibition was lowered in line with the reduction in the duration of paired-pulse inhibition and miniature IPSC (mIPSC) amplitude [40]. NL2 KO mice appear to exhibit a general reduction in inhibitory synaptic transmission, a phenomenon that was also demonstrated in acute cortical slices [33] and thalamocortical slices [36] (Table 2, NL2). Furthermore, in the retina of NL2 KO mice, GABA_A_ receptor levels were decreased, and ganglion cells in the NL2-deficient retina showed increased baseline activity and impaired amplitude response to light stimuli [72] (Table 2, NL2). Collectively, the results of imaging and electrophysiological studies indicated that NL2 potentially affects inhibitory synaptic transmission by regulating synaptic content in addition to synaptic structure.

NL2 also regulates the balance between glutamatergic and GABAergic synapse functions by selectively modulating inhibitory synaptic transmission. When NL2 was overexpressed in rat hippocampus by using adeno-associated virus injection, the mRNA level of GAD65 but not vGluT was increased [73] (Table 1, NL2). Imaging and electron microscopy revealed that in NL2 transgenic mice, the E/I ratio was decreased in the cortical region [74] (Table 1, NL2). Moreover, in NL2 transgenic mice, whole-cell patch-clamp recordings in the prefrontal cortex layer II/III pyramidal neurons showed increased basal inhibitory transmission [74] (Table 2, NL2). The functional GABA switch was abolished when NL2 was knocked down in cortical neurons [45], and the frequency of mIPSCs and miniature EPSCs (mEPSCs) was also decreased in cortical neurons transfected with an NL2 shRNA [45]. Notably, overexpression of the K-Cl cotransporter KCC2 partially rescued the reduction in mEPSC but not mIPSC [45], indicating a direct role of NL2 in regulating GABAergic synaptic transmission and an indirect role in regulating glutamatergic synapse function.

Similar to the function of NL2 described using the mouse model, NL2 was shown to be essential for synapse development and synaptic transmission in* Drosophila* [41].

### 2.3. NL3

Genetic mutations that lead to both partial deletion and point mutation of NL3 protein have been found in autism patients [7, 9, 75, 76]. In Japanese patients with autism, 4 novel substitutions were identified in NL3 and NL4 [15]. To investigate the functional consequences of the mutations, NL3 R451C, NL3 R704C, and NL3 KO mutations were introduced into mice.

In hippocampal neurons, NL3 overexpression caused an increase in the number of vGluT and vGAT puncta; conversely, vGluT puncta and spine numbers were decreased following NL3 knockdown [17]. However, excitatory synaptic transmission in hippocampal CA1 pyramidal neurons was unaffected after NL3 knockdown [77], but mIPSCs were increased in cultured neurons after NL3 overexpression [78]. To further elucidate the importance of NL3 in synaptic transmission, electrophysiological recordings were performed in the hippocampus, somatosensory cortex, and cerebellum of NL3 KO mice [38, 79]. The results showed a specific increase in mIPSCs and a decrease in mEPSC in the hippocampus [38] and a reduction in mEPSCs and impaired mGluR-mediated long-term depression (LTD) in the cerebellum [79], which indicated that NL3 was involved in basal synaptic transmission in these 2 brain regions.

The R451C mutation of NL3 is the most extensively investigated autism-associated NL3 mutation. In NL3 R451C KI mice, misfolding and trafficking defects in NL3 protein were detected and NL3 expression levels were lowered by 90% [34, 80]. Furthermore, in these KI mice, the expression of NL1 was decreased and that of the inhibitory-synapse proteins vGAT and gephyrin, but not vGluT, was increased [34]. Immunohistochemical analysis of the CA1, CA3, and somatosensory cortex regions revealed similar increases in vGAT density, in the absence of any change in vGluT density [34]. In the KI mice, dendritic complexity was increased, coupled with a substantial increase in dendritic branching, in stratum radiatum hippocampus [38]. Intriguingly, the R451C mutation in NL3 leads to distinct synaptic transmission changes in different brain regions: Whereas mEPSCs were increased only in the CA1 region of the hippocampus, mIPSCs were increased only in the somatosensory cortex [34, 38]. Furthermore, a large enhancement of LTP was detected, which could have occurred because of an alteration of NMDAR subunit composition and increased expression of NMDAR subunit 2B [38]. In the CA3 region in NL3 R451C KI mice, miniature GABAergic postsynaptic currents (mGPSCs) were also increased and the release of GABA was affected [44]; moreover, in the CA3 region, the frequency of network-driven giant depolarizing potentials (GDPs) was increased, indicating that the R451C mutation enhanced correlated network activity in the immature hippocampus [44].

Recently, paired whole-cell recordings were performed in the hippocampus of NL3 R451C mice, and the results revealed impaired GABAergic synaptic transmission at the parvalbumin- (PV-) positive basket-cell synapse, with a 70% reduction in IPSC amplitude and 20% reduction in IPSC success rate [42]; by comparison, GABAergic synaptic transmission at the cholecystokinin- (CCK-) positive basket-cell synapse exhibited a similar increase in IPSC amplitude but a higher IPSC success rate [42]. These findings showed that NL3 is critical for both GABAergic synaptic transmission in interneurons and interneuron connectivity to pyramidal neurons.

Interestingly, most of the aforementioned changes in synaptic transmission in pyramidal neurons or interneurons that were detected in NL3 R451C KI mice were not reproduced in NL3 KO mice [34, 38, 42]. The dissimilar electrophysiological behaviors recorded for these 2 types of NL3 mouse models suggest that the enhanced inhibitory synaptic transmission caused by NL3 R451C might be the consequence of a gain of protein function. Recently, in NL3 R451C KI mice, paired whole-cell recordings showed that GABAergic transmission was defective at synapses formed by PV-positive basket cells onto spiny neuron synapses in layer IV of the barrel cortex; however, no change was detected in the excitatory input to PV-positive basket cells or spiny neurons [46], suggesting that the primary targets of the NL3 mutation are the PV-positive basket cells.

Conversely, both NL3 R451C KI and NL3 KO mice showed increased IPSC amplitude and success rate at the CCK-positive basket-cell synapse [34, 38, 42]. However, in both of these mutant mice, IPSC amplitude and success rate at the same synapse failed to respond to AM251 (a CB1 receptor antagonist), which suggests that NL3 plays a crucial role in maintaining tonic endocannabinoid signaling in neurons [42]. Moreover, NL3 R451C KI, NL3 KO, and NL3 conditional deletion in D1-medium spiny neurons (D1-MSNs) all resulted in a reduction in IPSCs and an increase in the E/I ratio, which led to an enhanced rotarod learning behavior in the mutant mice [47]. Therefore, NL3 was also shown to be critical for synaptic transmission in the D1-MSNs of the nucleus accumbens, which mediates repetitive behavior.

Before the NL4 gene was identified in mouse [16], the NL4 autism-related mutation R704C [9] was introduced into the conserved site in NL3 [39]. Unlike the NL3 R451C mutation, the R704C mutation caused only a roughly 35% reduction in NL3 expression [39]. In NL3 R704C KI mice, examination of the expression of synaptic proteins revealed a selective increase in the levels of AMPAR subunits GluA1 and GluA3 [39]. Moreover, the AMPAR-mediated synaptic response was decreased, whereas the NMDAR- or GABAR-mediated synaptic response was unaltered [39]. In cultured hippocampal neurons, the NL3 R704C mutation caused a reduction in mEPSC frequency and an increase in the NMDA/AMPA ratio of receptor-mediated EPSCs, but NMDAR-dependent LTP was unchanged [39]. Collectively, these results showed that the R704C mutation in NL3 selectively impaired AMPAR-mediated synaptic transmission in the hippocampus.

### 2.4. NL4

NL4 is preferentially localized at glycinergic synapses and to a small extent to the GABAergic synapses [21]. When NL4 was knocked out, glycine receptor GlyR*α*1 numbers were substantially diminished in the retina and the decay in glycinergic mIPSCs was slowed, which indicated that some of the fastest glycinergic events were absent [21]. Because of the impaired inhibition, the latency in triggering the firing of retinal ganglion cells (RGCs) was shortened in multielectrode array recordings. Furthermore, in NL4 KO mice, electroretinogram recordings showed that the b-wave amplitude of the scotopic response was decreased, which indicated impaired bipolar cell activity [21]. Similar to NL2, NL4 can interact with collybistin and gephyrin [21, 31]. In NL2 KO retina, the number of NL4 clusters and NL4-containing inhibitory synapses were both increased, which suggested a functional relationship between NL2 and NL4 [21]. However, in visual processing, NL2 plays a more prominent role than does NL4: severely impaired visual acuity and contrast sensitivity were detected in NL2 KO mice, but not in NL4 KO mice [21].

The NL4 R87W mutation, which was found in 2 brothers with autism [13], impaired the glycosylation processing of NL4 and caused the protein to be retained in the ER [13]; this is similar to the effect of the NL3 R451C mutation [80]. However, unlike the NL3 R451C mutation, the NL4 R87W mutation abolished NL4-induced synapse formation [13]. Whereas NL4 overexpression in neurons caused a selective reduction in excitatory synaptic transmission, expression of the R87W mutant produced no change in synaptic transmission [13].

In* Drosophila*, NL4 was reported to be highly expressed in large ventral lateral clock neurons (l-LNvs), and in l-LNvs, NL4 was shown to be essential for sleep regulation: conditional depletion of NL4 in these neurons led to abnormal sleep, which could be rescued by specifically expressing NL4 in the l-LNvs of NL4 KO flies [43]. Moreover, in these KO flies, night sleep was decreased [43]. In NL4 KO flies, GABA currents were markedly diminished, indicating impaired GABA transmission [43]. Furthermore,* in vivo* co-IPs revealed an association between NL4 and RDL (the GABA_A_ receptor in* Drosophila*), and RDL clustering was substantially decreased in the l-LNvs of NL4 KO flies [43]; thus, NL4 likely regulates RDL clustering by associating with RDL. Collectively, these results showed that NL4 was essential for GABA_A_ receptor clustering and GABA transmission in* Drosophila*.

## 3. Effect of Synaptic Activity on Neuroligins

Synaptic activity is widely recognized to play a role in synaptogenesis and synapse maturation during brain development. NLs can influence synaptogenesis and play a critical role in both excitatory and inhibitory synaptic transmission. Conversely, synaptic activity is also required for NL functions. In addition, synaptic activity has been widely reported to modulate the surface expression, cleavage, and phosphorylation of NLs* in vitro* and* in vivo* and thereby affect NL-mediated functions.

### 3.1. Synaptic Activity Is Required for NL Synaptic Functions

Numerous studies have shown that NL1 is required for LTP and affects synaptic activity [35, 37, 50, 52–55]. Synaptic activity, mostly NMDAR activity, is also required for NL-induced changes in synaptic transmission. In hippocampal neurons, chronic treatment with AP5 or KN93 suppressed the NL1-induced increase in NMDAR- or AMPAR-mediated EPSCs, NMDA/AMPA ratio, and spine and synapse density [33]; moreover, when all neuronal network activity was chronically inhibited, NL2-induced increase in IPSCs was suppressed. The results showed that NL1- or NL2-induced increase in synaptic function depended on synaptic activity [33]. In cultured neurons, chronic application of AP5 also prevented NL1-overexpression-induced maturation of presynaptic boutons [57], which suggested that NL1 affected presynaptic maturation in an NMDAR activity-dependent manner. Furthermore,* in vivo* time-lapse imaging of neurons in* Xenopus* brain revealed that NL1-induced filopodial stabilization was reversed by the application of AP5, which increased the elimination rate of preexisting filopodia by 77% and markedly reduced the life time of filopodia; this suggested that NL1-mediated filopodial stabilization requires NMDAR activity [58]. Collectively, these results suggest that NL1 functions also depend on synaptic activity.

In contrast to synaptic activity-dependent regulation of NL1, activity-independent regulation of NL1 has also been reported [49, 51, 68]. In cultured neurons, APV/TTX treatment did not affect the level to which NL1/neurexin1*β* induced the recruitment of the AMPAR subunit GluA2 [49]. Furthermore, the effects of NL on excitatory synapses were also independent of excitatory synaptic activity. NL1 enhanced both NMDAR- and AMPAR-mediated EPSCs and NL3 enhanced AMPAR-mediated but not NMDAR-mediated currents. Application of NBQX and AP5 did not block the effects of NL1 and NL3 [51]. Moreover, in hippocampal neurons, chronic treatment with AP5 did not block NL1-overexpression-induced increase in synapsin and spine density [68].

### 3.2. Synaptic Activity Regulates NL Expression

Synaptic activity can also regulate the surface levels of NL1/3 in neurons. When LTP was chemically induced using forskolin/rolipram, NL1/3 showed a 100% increase in the surface level in acute hippocampal slices and a 50% increase in cultured hippocampal neurons [59]. By contrast, application of chemical LTD by using DHPG (an mGluR agonist) led to a corresponding reduction in NL1/3 surface levels [59]. In cultured hippocampal neurons, the use of live imaging and photoconductive stimulation showed that high-frequency activity increased the number of NL1 clusters and NL1 transport dynamics [56]. These findings revealed that the surface levels and the translocation of NL1/3 are regulated by synaptic activity.

### 3.3. Synaptic Activity Regulates NL Shedding

NL1 cleavage has been shown to be activity dependent. Ectodomain shedding of NL1 at the juxtamembrane stalk region was mainly mediated by ADAM10 and subsequently *γ*-secretase, and this generated a secreted N-terminal fragment of NL1 (NL1-NTF) and a membrane-tethered C-terminal fragment (CTF) [60]. In cortical neurons, treatment with AP5 or MK801 (NMDAR antagonists) reduced the NL1-NTF level in the media and MK801 treatment abolished the increase in NL1-NTF levels induced by glutamate treatment [60].

In cortical neurons, KCl-induced depolarization lowered the NL1 level at synapses and increased the level of NTF, whereas treatment with GM6001 (a broad-spectrum matrix metalloprotease inhibitor) abolished KCl-induced loss of NL1 [61]. Moreover, NL1-NTF levels were decreased following TTX treatment but increased markedly after treatment with bicuculline (BCC, a GABA_A_-receptor antagonist) and 4-AP or NMDA [61], and both AP5 and CaMKII-inhibitor treatment abolished KCl-induced increase in NL1-NTF levels [61]. By contrast, binding to neurexin1*β* enhanced the cleavage of NL1 and increased NL1-NTF levels [60].

NL1 cleavage was also shown to be regulated by synaptic activity* in vivo*. In the live mouse brain, status seizure induced by injection of M-receptor-agonist pilocarpine caused an increase in NL1-NTF levels in the forebrain [60] and the hippocampus [61]. In a dark-rearing experiment, mice were subjected to 5 days of dark rearing (P21–P26) and then re-exposed to light for 2 h; this re-exposure induced rapid synaptic remodeling in the visual cortex, and the mice showed enhanced cleavage of NL1 and an increased level of NL1-NTF in the visual cortex [61]. These results suggest that the increased excitatory activity enhanced NL1 cleavage* in vivo*.

Activity-dependent cleavage of NL1 was demonstrated to negatively regulate the spinogenic activity of NL1 and subsequently regulate synaptic transmission. In CA1 pyramidal neurons, the results of glutamate-uncaging experiments revealed that NL1 cleavage occurs only in activated dendritic spines [61]. In dentate granule cells, the overexpression of NL1 or NL1-CTF, but not NL1 intracellular domain, increased spine density [60], and in hippocampal neurons, overexpression of cleavage-deficient NL1 but not WT NL1 substantially increased spine density [60]. Furthermore, electrophysiological studies showed that NL1 cleavage reduced excitatory neurotransmission by lowering the probability of neurotransmitter release, which was revealed by a reduction in both EPSC frequency and eEPSC amplitude and an increase in eEPSC paired-pulse ratio [61]. Conversely, blocking NL1 cleavage increased presynaptic release probability [61].

### 3.4. Synaptic Activity Regulates NL Phosphorylation

In addition to activity-dependent regulation of NL function and shedding, synaptic activity-induced NL1 phosphorylation specifically at T739 by CaMKII has been demonstrated. In cortical neurons, NL1 T793 phosphorylation was modulated by synaptic activity: treatment with the GABA_A_-receptor antagonist BCC enhanced NL1 phosphorylation, which was efficiently blocked when neurons were pretreated with AP5 and NBQX; this indicated that NL1 phosphorylation was dynamically regulated by synaptic activity [62]. BCC treatment also caused a reduction in the total NL1 level [62], which likely was the result of NL1 ectodomain shedding as previously reported [60, 61]. In cortical neurons, shRNA-mediated lowering of CaMKII levels by 75% led to a 60% reduction in the level NL1 T739 phosphorylation in the absence of any change in the total NL1 level [62]. NL1 T739 phosphorylation was also regulated by synaptic activity* in vivo* in an experience-dependent manner. In a dark-rearing experiment, NL1 T739 phosphorylation was lower in dark-reared mice than in light-reared mice, and following re-exposure to light for 2 h after 5 days of dark-rearing, the mice showed an increase in NL1 T739 phosphorylation [62]. The phosphorylation state was found to regulate the surface expression level of NL1 and then maintain the recruitment of critical synaptic proteins such as vGlut and PSD-95 [62]. In hippocampal neurons, the surface expression of the NL1 T739A mutant (phosphorylation-deficient mutant) was markedly diminished, which suggested that NL1 trafficking or stabilization at the plasma membrane was regulated by its phosphorylation [62]. Moreover, the expression of the NL1 T739A mutant, unlike that of NL1, did not enhance mEPSC frequency [62]. Taken together, these results suggest that activity-dependent NL1 T739 phosphorylation is critical for both NL1 surface expression and NL1-mediated synaptic function.

## Figures and Tables

**Table 1:** Neuroligin-induced changes in synaptic protein expression.

	Knockout	Acute knockdown	Knock-in	Acute overexpression	Transgenic	Reference
NL1	**CA1 HP**: vGluT (no), vGAT (no) **CA3 HP**: vGluT (no), vGAT (no) **Layer 2/3 pyramidal neurons:** spine density (no) **WB of whole brain**: synapsin-1a/b (+), NL3 (+), *α*/*β*-neurexin (−), PSD-95 (no), vGluT (no), vGAT (no) **Cultured cortical neurons**: spine density (no) **WB of HP**: GluA1 (no), GluA2 (−), GluN2A (−), GluN2B (−), GluN1 (−)	**DG HP**: spine density (−) **LA**: spine density (no) **Layer 2/3 pyramidal neurons: **spine density (−) **Cultured HP neurons**: vGluT (−), vGAT (−), spine number (−)		**Cultured HP neurons**: spine number (+), GluN1 (+), GluN2A (+), GluN2B (+), PSD95 (+), vGluT (+), vGAT (+), vGluT : vGAT (no), synapsin (+), GluA1 (no) GluA2/3 (+), bassoon (+), synaptophysin (+) **Cultured HP neurons (NL1 R473C overexpression)**: spine number (−), synapsin (−)	**NL1 Tg mice**: **CA1 HP**: vGluT (+), vGAT (+), vGluT/PSD-95 cocluster (+), vGAT/gephyrin cocluster (no), spine density (+), spine length (no), spine head (+), spine neck (−), asymmetric synapse (+), symmetric synapse (no), E/I ratio (+) **WB**: gephyrin (+), PSD-95 (+), vGluT (+), vGAT (+), GluA2/3 (−), NL3 (+), GluA1 (+)	[17, 29, 32, 33, 35, 37, 52–55, 57, 66–68]

NL2	**CA1 HP**: vGAT (−), vGluT (no), synaptophysin (no), asymmetric synapse (no), symmetric synapse (no), VIAAT (no), gephyrin in pyramidale (−), gephyrin in radiatum (no) **CA3 HP**: vGAT (−), vGluT (no), synaptophysin (no) **GCL**: gephyrin (−), VIAAT (no), GABA_A_R*γ*2 subunit (−) **ML**: no change in gephyrin, VIAAT, or GABAR_A_ *γ*2 **Somatic region of cultured neurons**: cytoplasmic gephyrin (+), postsynaptic gephyrin (−) **WB of whole brain**: synaptobrevin2 (+), no change in several other synaptic proteins **Retina IPL**: GABA_A_ *α*1 (no), GABA_A_ *α*3 (−), GABA_A_ *γ*2 (−), gephyrin (no), GlyR (no)	**HP cultured neurons**: vGluT (−), vGAT (−), spine number (−)		**Cultured HP neurons**: vGluT (+), vGAT (+), vGluT : vGAT puncta (−), spine density (+), synapsin (no) **Dorsal HP (mRNA expression)**: GAD65 (+), GAD67 (no), vGluT (no), gephyrin (no) **Ventral HP (mRNA expression):** GAD65 (no), GAD67 (no), vGluT (no), gephyrin (no)	**NL2 Tg mice:** **mPFC**: vGluT (+), vGAT (+), E/I ratio (−) **EM of mPFC**: total synapse density (+), symmetric synapse (+), asymmetric synapse (no), E/I ratio (−) **WB of whole brain**: syntaxin (+), vGluT (+), vGAT (+), NL3 (−), NL1 (no), gephyrin (no), PSD-95 (no)	[17, 31, 33, 40, 45, 71–74]

NL3	**WB of brain**: NL1 (−), vGAT (no), gephyrin (no), vGluT (no) **CA1 HP**: vGAT (no), vGluT (no), synaptophysin (no) **CA3 HP**: vGAT (no), vGluT (no), synaptophysin (no) **SSC**: vGAT (no), vGluT (no)	**HP cultured neurons**: vGluT (−), vGAT (no), spine number (−)	**NL3 R451C KI**: **CA1 HP**: vGAT (+), vGluT (no), synaptophysin (no), spine density (no), stratum radiatum branch points (+) **CA3 HP and SSC**: vGAT (+), vGluT (no) **WB of whole brain**: vGAT (+), gephyrin (+), vGluT (no), NL1 (−), NL3 (−) **WB of HP**: PSD-95 (+), vGluT (no), vGAT (no), NL3 (−), SAP-102 (+), GluA1 (no), NR1 (no), NR2A (+), NR2B (+) **EM from layer2/3 of SSC**: synapse number (no), asymmetric/symmetric (no), PSD length (no) **EM from stratum radiatum CA1 HP**: presynaptic nerve terminal size (−), vesicle number (−), spine size (−), PSD length (no), docked vesicle number (no) **NL3 R704C KI**: **WB of whole brain**: GluA1 (+), GluA3 (+), NL3 (−), no change in many other synaptic proteins **CA1 HP**: synaptophysin (no), vGluT (no), vGAT (no) **CA3 HP**: synaptophysin (no), vGluT (no), vGAT (no) **SSC**: synaptophysin (no), vGluT (no), vGAT (no)	**Cultured HP neurons**: vGluT (+), vGAT (+), vGluT : vGAT (no), synapsin density (+) **Cultured HP neurons (NL3 R704C overexpression)**: synapsin (+), synapse density (+)		[17, 34, 38, 39]

NL4	**Retina:** GlyR*α*1 (−), GlyR*α*2 (no), GlyR*α*3 (no), GlyR*α*4 (no)			**Cultured HP neurons**: spine (+), synapse density (+) **Cultured HP neurons (NL4 R87W overexpression)**: spine (no), synapse density (no)		[13, 21]

NL1/2/3	**PBC**: vGluT (+), VIAAT (−) **IOM**: vGluT (+), VIAAT (−) **NH:** vGluT (no), VIAAT (no) **WB:** vGluT1 (−), complexin2 (−), *α*SNAP (−), synaptobrevin (−), synaptophysin (−), synaptotagmin (−), KCC2 (−), GluN1 (−), no change in many other synaptic proteins **Cultured HP neurons**: synapsin (no), PSD-95 (no) **Cultured cortical neurons**: PSD length (no), synaptic cleft width (no), docked vesicle number (no)	**Cultured HP neurons**: vGluT (−), vGAT (−), spine number (−)				[17, 48]

The synaptic protein-expression changes summarized in the table were all measured in immunohistochemical assays performed on brain slices unless specified otherwise. Other assays: WB, Western blot; EM, electron microscopy. Brain regions: DG, dentate gyrus; LA, lateral amygdala; mPFC, medial prefrontal cortex; HP, hippocampus; GCL, granule cell layer; SSC, somatosensory cortex; PBC, pre-Botzinger complex; ML, molecular layer; NH, hypoglossal nucleus. Detected changes: +, increase; −, decrease; no, no significant change.

**Table 2:** Neuroligin-induced changes in synaptic transmission.

	Knockout	Acute knockdown	Knock-in	Acute overexpression	Transgenic	Reference
NL1	**CA1 HP**: NMDA EPSC (−), AMPA EPSC (no), NMDA/AMPA ratio (−) **SSC**: IPSC amplitude (no) **Cortical slices**: IPSC amplitude (no) **DG HP**: slope of fEPSC (−), LTP (−), field EPSP-population spike (E-S) coupling (+) **Amygdala**: NMDA EPSC (−), AMPA EPSC (no), NMDAR/AMPAR ratio (−), thalamic STD-LTP (−), cortical STD-LTP (no) **Str.corticostriatal synapse**: NMDA/AMPA ratio (−) **Cortical layer 2/3 pyramidal neurons**: AMPA uEPSC (no), NMDA uEPSC (−), NMDA/AMPA ratio (−)	**CA1 HP**: eliminated young but not adult hippocampal LTP **DG HP**: eliminated adult but not young hippocampal LTP, young and adult baseline current (−) **Amygdala**: NMDA EPSC (−), AMPA EPSC (no), NMDA/AMPA ratio (−), LTP (−) **Cortical layer 2/3 pyramidal neurons**: mEPSC amplitude (no), mEPSC frequency (−), AMPA uEPSC (no), NMDA uEPSC (−), NMDA/AMPA ratio (−)		**Cultured HP neurons**: mEPSC frequency (+), mEPSC amplitude (no), mIPSC frequency (+), mIPSC amplitude (no), AMPA EPSC (+), NMDA EPSC (+), NMDA/AMPA ratio (+) **Cultured HP neurons (NL1 R473C overexpression)**: AMPA EPSC (−), NMDA EPSC (−), NMDA/AMPA ratio (no)	**NL1 Tg mice**: **CA1**: LTP (−)	[29, 33, 35, 37, 50, 52–55, 67]

NL2	**Brainstem slices**: eIPSC amplitude (−), eEPSC amplitude (no), sIPSC frequency (−), sIPSC amplitude (−), sEPSC (no), mIPSC (−), mEPSC (no) **CA1**: GABAergic sIPSC (−), GABAergic mIPSC (−) **DG**: population spike amplitude (+), threshold frequency for epileptiform discharge induction (−), duration of PPI (−), mIPSC amplitude (−), LTP (no) **Cortical slices**: IPSC amplitude (−) **SSC**: FS interneurons: IPSC amplitude (−); SP interneurons: IPSC amplitude (no) **RGC**: rate of basal action potential firing (+), STA latency (−), amplitude of response to light stimuli (−)	**Cortical neurons**: mIPSC frequency (−), mEPSC frequency (−)		**Cultured HP neurons**: IPSC amplitude (+), AMPA or NMDA EPSC (no), NMDA/AMPA ratio (no)	**NL2 Tg mice:** **PFC**: mIPSC frequency (+), mIPSC amplitude (no), mEPSC (no) **RGC**: sIPSC (no)	[31, 33, 36, 40, 45, 72, 74, 81]

NL3	**CA1 HP**: mEPSC frequency (−), mIPSC frequency (+), NMDA/AMPA ratio (no), fEPSP slope (no), LTP (no) **SSC**: eEPSC amplitude (no), eIPSC amplitude (no) **Cerebellum**: mEPSC (−), mGluR-mediated LTD (−) **PV-pyramidal neurons**: IPSC amplitude (no), IPSC success rate (no) **CCK-pyramidal neurons**: IPSC amplitude (+), IPSC success rate (+) **NL3 conditional deletion in DR D1-MSNs**: mIPSC (−), E/I ratio (+)	**CA1 HP**: AMPA EPSC (no), NMDA EPSC (no), NMDA/AMPA ratio (no), eliminated young but not adult hippocampal LTP **DG**: young or adult hippocampal LTP (no), adult base line current (−)	**NL3 R451C KI mice**: **CA1:** mEPSC (+), mIPSC (no), LTP (+), fEPSC slope (+), NMDA/AMPA ratio (+) **CA3**: mGPSC frequency (+), mGPSC amplitude (no), GDP frequency (+), mEPSC frequency (P27-35) (+) **SSC:** NMDA/AMPA ratio (no), mEPSCs (no), mIPSC frequency (+), mIPSC amplitude (no), eEPSC amplitude (no), eIPSC amplitude (+) **PV-pyramidal neurons**: IPSC amplitude (−), IPSC success rate (−) **CCK-pyramidal neurons**: IPSC amplitude (+), IPSC success rate (+) **PV-spiny neuron synapse in layer IV cortex**: IPSC amplitude (−), E/I ratio (+), EPSC amplitude (no) **NL3 R704C KI mice**: **CA1 HP**: mEPSC frequency (−), mEPSC amplitude (no), mIPSC (no), fEPSP slope (−), PPF (no) **HP slices**: NMDA/AMPA EPSC ratio (+), NMDAR-dependent LTP (no) **Cultured HP neurons**: AMPA EPSC amplitude (−), NMDA EPSC amplitude (no), GABA IPSC amplitude (no)	**Cultured HP neurons**: mIPSC (+) **Cultured HP neuron (NL3 R471C overexpression)**: mIPSC amplitude (+), mIPSC frequency (no)		[34, 38, 39, 42, 44, 46, 47, 54, 77–79]

NL4	**RGC**: glycinergic mIPSC (no), GABAergic mIPSC (no), decay time of glycinergic mIPSC (+), RGC response latency (−), latency in triggering action potentials (−), b-wave amplitude of scotopic response (−) ***Drosophila* l-LNvs:** GABA current (−)			**Cultured HP neurons**: mEPSC frequency (−), mEPSC amplitude (no), mIPSC (no), evoked EPSC amplitude (−), evoked IPSC amplitude (no), EPSC charge (−) **Cultured HP neurons (NL4 R87W overexpression)**: no change in mEPSC, mIPSC, EPSC, IPSC		[13, 21, 43]

NL1/2/3	**PBC slices**: GABAergic/glycinergic mPSC and sPSC frequency (−), glutamatergic mPSC and sPSC frequency (−)	**Cultured HP neurons**: mEPSC amplitude (−), mIPSC (−)				[17, 48]

The electrophysiological studies summarized in this table were all conducted on brain slices unless specified otherwise, and the listed changes in synaptic transmission were both amplitude and frequency changes unless specified otherwise. Brain regions: DG, dentate gyrus; LA, lateral amygdala; RVLM, rostral ventrolateral medulla; SSC, somatosensory cortex; PFC, prefrontal cortex; mPFC, medial prefrontal cortex; DS, dorsal striatum; D1-MSNs, dopamine D1 receptor-expressing medium spiny neurons; HP, hippocampus; RGC, retinal ganglion cell; PBC, pre-Botzinger complex; l-LNvs, large ventral lateral neurons; FS: fast-spiking; SP: somatostatin-positive. PPF: paired-pulse facilitation. Detected changes: +, increase; −, decrease; no, no significant change.
